# Sox9 transcriptionally regulates Wnt signaling in intestinal epithelial stem cells in hypomethylated crypts in the diabetic state

**DOI:** 10.1186/s13287-017-0507-4

**Published:** 2017-03-09

**Authors:** Can-Ze Huang, Ji-Hao Xu, Wa Zhong, Zhong-Sheng Xia, Si-Yi Wang, Di Cheng, Jie-Yao Li, Ting-Feng Wu, Qi-Kui Chen, Tao Yu

**Affiliations:** 1 Department of Gastroenterology, Sun Yat-Sen Memorial Hospital, Sun Yat-Sen University, 107 Yan Jiang Xi Road, Guangzhou, Guangdong 510120 China; 2 Guangdong Provincial Key Laboratory of Malignant Tumor Epigenetics and Gene Regulation, Sun Yat-Sen Memorial Hospital, Sun Yat-Sen University, 107 Yan Jiang Xi Road, Guangzhou, Guangdong 510120 China

**Keywords:** Sox9, Wnt signaling, Intestinal epithelium stem cells, DNA methylation, Diabetes mellitus

## Abstract

**Background:**

Distinctive structures called crypts harbor intestinal epithelial stem cells (IESCs) which generate progenitor and terminally differentiated cells in the intestinal epithelium. Mammalian IESCs and their daughter cells require the participation of DNA methylation and the transcription factor Sox9 for proliferation and differentiation. However, the association between Sox9 and DNA methylation in this process remains elusive.

**Methods:**

The DNA methylation of small intestinal epithelial crypts in *db/db* mice was detected via combining methylated DNA immunoprecipitation with microarray hybridization. DNA methylation of Sox9 promoter in crypts and IESCs was validated using bisulfite sequence analysis. The target sequence of the transcription factor Sox9 in IESCs was investigated via chromatin immunoprecipitation (ChIP) combined with deep sequencing (ChIP-seq).

**Results:**

Increased *Sox9* expression is accompanied by the loss of methylation in its promoter in IESCs. Sox9 targets the enhancers of the Wnt signaling pathway-related genes. Sox9 predominantly acts as a transcriptional activator at proximal enhancers of *Wnt4*, *Tab2*, *Sox4*, and *Fzd8*, but also functions as a potential transcriptional inhibitor at a distant enhancer of *Cdk1*. Lack of Sox9 transcriptional activation in specific repressors of the Wnt signaling pathway leads to the loss of intrinsic inhibitory action and ultimately produces overactivation of this pathway in *db/db* mice.

**Conclusions:**

Our study sheds light on the connections among DNA methylation, transcription factor modulation, and Wnt signaling in IESCs in the diabetic state. Hypomethylation in the Sox9 promoter is correlated to increased Sox9 expression in *db/db* IESCs. Although there is increased expression of Sox9 in *db/db* IESCs, the loss of Sox9 transcriptional activation in specific repressors of the Wnt signaling pathway might result in abnormalities in this pathway.

**Electronic supplementary material:**

The online version of this article (doi:10.1186/s13287-017-0507-4) contains supplementary material, which is available to authorized users.

## Background

The small intestinal epithelium has a distinctive organization, with intestinal epithelial stem cells (IESCs) harbored at the base of crypts. IESCs produce progenitors and differentiated cells. The length of the crypt-villus unit is significantly increased in 4- to 10-week-old diabetic mice, with increased proliferation labeling indexes in the small intestine [1]. Furthermore, the proportion of cells positive for leucine-rich repeat-containing G protein-coupled receptor 5 (Lgr5), which represent bona fide stem cells limited to the IESCs [2], is markedly increased in the crypts of diabetic mice [3]. In general, the signaling most closely connected to the abnormal proliferation and differentiation of IESCs is Wnt signaling [2]. Enhanced intestinal epithelial cell proliferation in diabetes mellitus (DM) has been demonstrated not only in experimental diabetic mice but also in recent clinical trials [4, 5]. Excessive activation of Wnt signaling in stem cells increases intestinal epithelial cell proliferation and even promotes formation of adenomas [6].

A controversial transcription factor (TF), Sry (sex determining region Y)-box 9 (Sox9), is correlated with the Wnt signaling pathway and plays a significant role in intestinal crypts. Traditionally, Sox9 is considered to function as a negative transcriptional regulator of Wnt signaling [7, 8]. However, recent studies using Sox9 chromatin immunoprecipitation (ChIP) combined with deep sequencing (ChIP-seq) analysis have reported that Sox9 can activate canonical Wnt/β-catenin signaling in hepatocellular carcinoma [9] and prostate cancer [10]. In another *Sox9* knockout mouse model, Wnt target genes appear to be unaltered in telogen hair follicles [11]. To date, it remains unclear how Sox9 modulates IESCs via Wnt signaling pathways.

A DNA methyltransferase 1 (*Dnmt1*) deletion mouse model has been reported to show significantly expanded crypts, increased Sox9 expression, and reduced DNA methylation in the crypts of the small intestine [12]. Dnmt1-mediated developmentally programmed epigenetic mechanisms are involved in the regulation of IESC function [13, 14]. Moreover, DNA methylation is required for the modulation of stem cell proliferation and differentiation in the small intestine. In the present study, we identify morphological and physiological changes in *db/db* mice, a well-established animal model of type 2 DM [15] that is similar to *Dnmt1*-deletion mice. More importantly, increased Sox9 expression is accompanied by hypomethylation of promoters in IESCs. Based on ChIP-seq analysis in IESCs, among Sox9 binding sites related to Wnt signaling, Sox9 targets the enhancers of genes related to the Wnt signaling pathway. It is not only predominantly acting as a transcriptional activator at proximal enhancers but also as a potential transcriptional inhibitor at a distant enhancer. Lack of Sox9 transcriptional activation in specific repressors of the Wnt signaling pathway eventually produces abnormal signaling in this pathway.

## Methods

### Mice

BKS.Cg-Dock7^m^ +/+ Lepr^*db*^/JNju (*db/db*) male mice were obtained from the Model Animal Research Center of Nanjing University (Nanjing, Jiangsu, China) and were maintained under a 12-h light/12-h dark cycle in a specific pathogen-free animal facility. The experiments were conducted using 16-week-old male homozygous mice, which were maintained for 8 weeks with hyperglycemia (random blood glucose level ≥16.7 mmol/l) prior to euthanasia. Identical genetic background BKS heterozygote *db/+* mice (random blood glucose level <11.1 mmol/l) were used as the controls. All experiments with mice were approved by the Animal Care Committee of Sun Yat-Sen University (Permit Number: 201412000091).

### Isolation of intestinal crypts and villus fractions

The upper half of the small intestine (from the duodenal end to the middle of the intestine) was dissected out from the mice and sliced longitudinally to expose the crypts and villi, in ice-cold phosphate-buffered saline (PBS) (with Mg^2+^/Ca^2+^). The intestine was subsequently incubated in ice-cold dissociation reagent #1 (47 ml DPBS without Mg^2+^/Ca^2+^; 3 ml 0.5 M EDTA (Sigma, St. Louis, MO, USA); 75 μl 1 M DTT (Sigma)) in a 15-ml tube and embedded in ice for 20 min, followed by the addition of dissociation reagent #2 (47 ml DPBS, 3 ml 0.5 M EDTA) and incubation at 37 °C for 10 min. Following incubation, each tube containing intestine was shaken for 30 s to release the epithelium from the basement membrane. The remaining intestinal tissue was removed, and cells shed into dissociation reagent #2 were collected and labeled as fraction 1. The solution containing dissociation reagent #2 was filtered through a 70-μm nylon cell strainer (BD Falcon, Corning, New York, NY, USA). The tissue retained on the filter, which consisted of villi, was stored in PBS (Mg^2+^/Ca^2+^) on ice (fraction 2). The incubation, shaking, and straining steps were repeated until eight fractions were collected. Fractions 3–6 comprised pure villus tissue identified as differentiated cells, and fractions 7 and 8 isolated as the flow-through from the cell strainer comprised pure crypt tissue. Pure crypt tissues were confirmed by traditional microscope.

### Promoter methylation microarray

Crypts were collected from six independent mice, with the control *db/+* mice designated C1, C2, and C3, and the diabetic *db/db* mice designated D1, D2, and D3. Immunoprecipitation of methylated DNA was performed using Biomag™ magnetic beads (Bangs Laboratories, IN, USA) coupled with a mouse monoclonal antibody against 5-methylcytidine. The total input and immunoprecipitated DNA were labeled with Cy3- and Cy5-labeled random 9-mers, respectively, and hybridized to ArrayStar Mouse RefSeq Promoter Arrays, which consisted of a multiplex slide with four identical arrays per slide; each array contained 22,327 well-characterized RefSeq promoter regions, from approximately −1300 bp to +500 bp of transcription start site (TSS), covered by 180,000 probes. Scanning was performed using an Agilent Scanner G2505C (Agilent Technologies, Santa Clara, CA, USA).

When comparing the differential methylation enrichment peaks (DMEPs) between two groups, we averaged the log2-ratio values for each group and calculated the M’ value using the following equation:

$$ \mathrm{M}' = \mathrm{Average}\ \left( \log 2\ {\mathrm{MeDIP}}_{db/ db}/{\mathrm{Input}}_{db/ db}\right)\ \hbox{--}\ \mathrm{Average}\ \left( \log 2\ {\mathrm{MeDIP}}_{db/+}/{\mathrm{Input}}_{db/+}\right). $$

The DMEPs are filtered according to the following criteria:

i) At least one of the two groups has a median (log2 MeDIP/Input) ≥0.3 and M’ >0.
ii) At least half of the probes in a peak may have a coefficient of variability (CV) ≤0.8 in both groups.

The dataset is available in the GEO database under the accession number GSE87044.

### Collection of IESCs

The crypts obtained as described above were resuspended in 10 ml Hank’s balanced salt solution (HBSS) containing 8 mg dispase (Gibco, Carlsbad, CA, USA). The cell solution was incubated for 10 min at 37 °C, with vigorous shaking every 2 min, followed by the addition of 10% fetal bovine serum (FBS) and 50 μl 10 mg/ml DNase (10 mg/ml; Roche, Basel, Switzerland). The solution was sequentially passed through 70- and 40-μm filters to collect the cell suspension. Single-cell suspension was confirmed under microscope. The primary antibodies Lgr5 (Abcam, Cambridge, MA, USA) and CD45-FITC (BD Pharmingen, San Jose, CA, USA) and the secondary antibody goat anti-rabbit IgG-PE (BD Pharmingen) were used for fluorescence-activated cell sorting (FACS). Lgr5^hi^CD45^neg^ cells, identified as IESCs, were sorted using a FACSVerse (BD Bioscience, San Jose, CA, USA) machine. All data analyses were performed using FlowJo V10 software (Treestar, USA).

Due to limited cell quantities when using FACS, single-cell suspensions were also sorted by magnetic bead-activated cell sorting (MACS; BD IMag™ Streptavidin Particles Plus-DM; BD Biosciences). Cells were resuspended in cell-staining buffer at a concentration of 2 × 10^7^ cells per ml; then, the biotinylated antibody Lgr5-Biotin (20 μl per 2 × 10^7^ cells; Biorbyt, Cambridge, UK) was added, followed by incubation on ice for 30 min. The labeled cells were subsequently washed with buffer, and 50 μl magnetic nanoparticles were added and incubated at 6–12 °C for 30 min. Finally, the labeled cells were washed with buffer three times using a magnetic device. The cells collected, predominantly IESCs, were used for Western blot analysis.

The IESCs sorted by FACS or MACS were validated for cell purity by quantitative real-time polymerase chain reaction (qRT-PCR).

### RNA extraction and quantitative real-time RT-PCR

Total RNA from samples was extracted using TRIzol® Reagent (Life Technologies, Carlsbad, CA, USA), and the concentration was calculated from the absorbance at 260 nm as determined using an ND-2000 instrument (NanoDrop Technologies, Wilmington, DE, USA), followed by reverse transcription with PrimeScript™ RT Master Mix (TaKaRa Bio, Kusatsu, Shiga, Japan). qRT-PCR was performed on a CFX Connect™ real-time PCR detection system (Bio-Rad, Hercules, CA, USA) using SYBR® Premix Ex Taq™ (TaKaRa Bio, Kusatsu, Shiga, Japan). The primers are shown in Additional file 1 (Table S1). Data were analyzed using the △△Ct method with β-actin as an internal control. The qRT-PCR experiments were performed in sextuplicate using independent samples.

### Western blot analysis

Isolated crypts or IESC samples were incubated in RIPA buffer (Thermo Scientific, Carlsbad, CA, USA). The protein samples were separated by 12% SDS-PAGE and transferred to PVDF membranes. The membranes were probed overnight at 4 °C with the following primary antibodies: rabbit anti-mouse Dnmt1 antibody (1:2000; Abcam, Cambridge, MA, UK), rabbit anti-mouse Dnmt3a antibody (1:1000; Abcam), rabbit anti-mouse Sox9 antibody (1:1500; Abcam), and rabbit anti-mouse β-actin antibody (1:2000; Cell Signaling Technology, Danvers, MA, USA). The data were analyzed using relative intensity with β-actin as the internal control. The Western blot experiments were performed in three repetitions using independent samples.

### Cell culture and transfection

The cell lines HCT116, Caco-2, DLD-1, and HT-29 were purchased from the Institute of Biochemistry and Cell Biology of the Chinese Academy of Sciences (Shanghai, China). Cells were cultured in RPMI 1640 medium or DMEM (Gibco, Carlsbad, CA, USA) supplemented with 10% FBS, 100 U/ml penicillin, and 100 mg/ml streptomycin (Gibco) in humidified air at 37 °C with 5% CO_2_. For *SOX9* overexpression and knockdown, SOX9-pcDNA (SOX9 expression plasmid) and SOX9 small interfering RNA (siRNA) were respectively transfected using Lipofectamine® 3000 Transfection Reagent (Invitrogen, Carlsbad, CA, USA). For qRT-PCR and Western blot analysis, cells were collected at 48 h and 72 h after transfection, respectively. The qRT-PCR and Western blot experiments were performed in quadruplicate using independent samples.

### Luciferase reporter assay

The human embryonic kidney (HEK) 293FT cell was used to transfect in 96-well plates. For each well, 300 ng of the pCMV-SOX9-3FLAG-SV40-Neomycin construct or the negative control pCMV-3FLAG-SV40-Neomycin backbone were transfected in combination with 50 ng of firefly luciferase reporter constructs and 50 ng of the pDC315-3FLAG-SV40-renilla luc vector (Gene Chem, Shanghai, China) using as the renilla luciferase reference. Luciferase activity was measured after 48 h using the Dual-Luciferase® Reporter Assay System (Promega, Madison, WI, USA) according to the manufacturer’s protocol.

### DNA extraction and bisulfite sequence analysis

Genomic DNA was extracted from crypts or IESCs using a Quick-DNA™ Universal Kit (Zymo Research, Tustin, CA, USA). DNA concentration was calculated from the absorbance at 260 nm with an ND-2000 instrument (NanoDrop Technologies, Wilmington, DE, USA). Purified genomic DNA was subjected to bisulfite conversion using an EZ DNA Methylation-Gold™ Kit (Zymo Research, Tustin, CA, USA). Converted DNA was PCR amplified using primer sets designed to target the methylated peaks of *Dnmt3b* and *Sox9*. A two-step amplification method was performed using a TaKaRa EpiTaq™ HS DNA polymerase system (TaKaRa Bio, Kusatsu, Shiga, Japan) under the following conditions: 98 °C for 2 min and 40 cycles of 98 °C for 10 s, 55 °C for 30 s, and 72 °C for 30 s. PCR products were subcloned into bacteria using a pMD™19 T-Vector Cloning Kit (TaKaRa Bio, Kusatsu, Shiga, Japan), and 10 colonies per group were sequenced using a BigDye Terminator v3.1 Cycle Sequencing kit and a 3730xl DNA Analyzer (Applied Biosystems, Carlsbad, CA, USA). The PCR primer sets are described in Additional file 1 (Table S2).

### ChIP-seq analysis

Independent immunoprecipitations were performed on FACS-sorted populations. Due to the limited numbers of IESCs in individual mice, a pooled cohort of four *db/db* mice was collected as the *db/db* group, and cells from five *db/+* mice were collected as the control group. Immunoprecipitated DNA was obtained using an EZ-Magna ChIP™ HiSens kit (Millipore, Temecula, CA, USA). Chromatin was sonicated into 200–500 bp fragments using the BioRuptor UCD-200 (Diagenode, Seraing, Belgium). Immunoprecipitation was performed using 4 μg rabbit polyclonal anti-mouse Sox9 antibody (Abcam, Cambridge, MA, UK) and 200 μl chromatin for each sample. Paired-end sequencing was performed on an Illumina HiSeq 2000 (50-bp reads). ChIP-seq reads were aligned to the mouse genome (mm9, build 37), and peaks were called with Model-based analysis of ChIP-Seq [16]. The Integrative Genomics Viewer was used to visualize signal tracks. The MEME software suite was applied to the sequences under the Sox9 peaks (200 bp around summit) to identify enriched motifs. The dataset is available in the GEO database under the accession number GSE87583.

### Statistical analysis

To establish the significance between two groups, comparisons were performed using unpaired two-tailed Student’s *t* tests in Prism 5 (GraphPad software). For all statistical tests, the 0.05 level of confidence was considered statistically significant.

## Results

### Decreased DNA methylation of promoter regions in crypts was correlated to the diabetic state

**Fig. 1:**
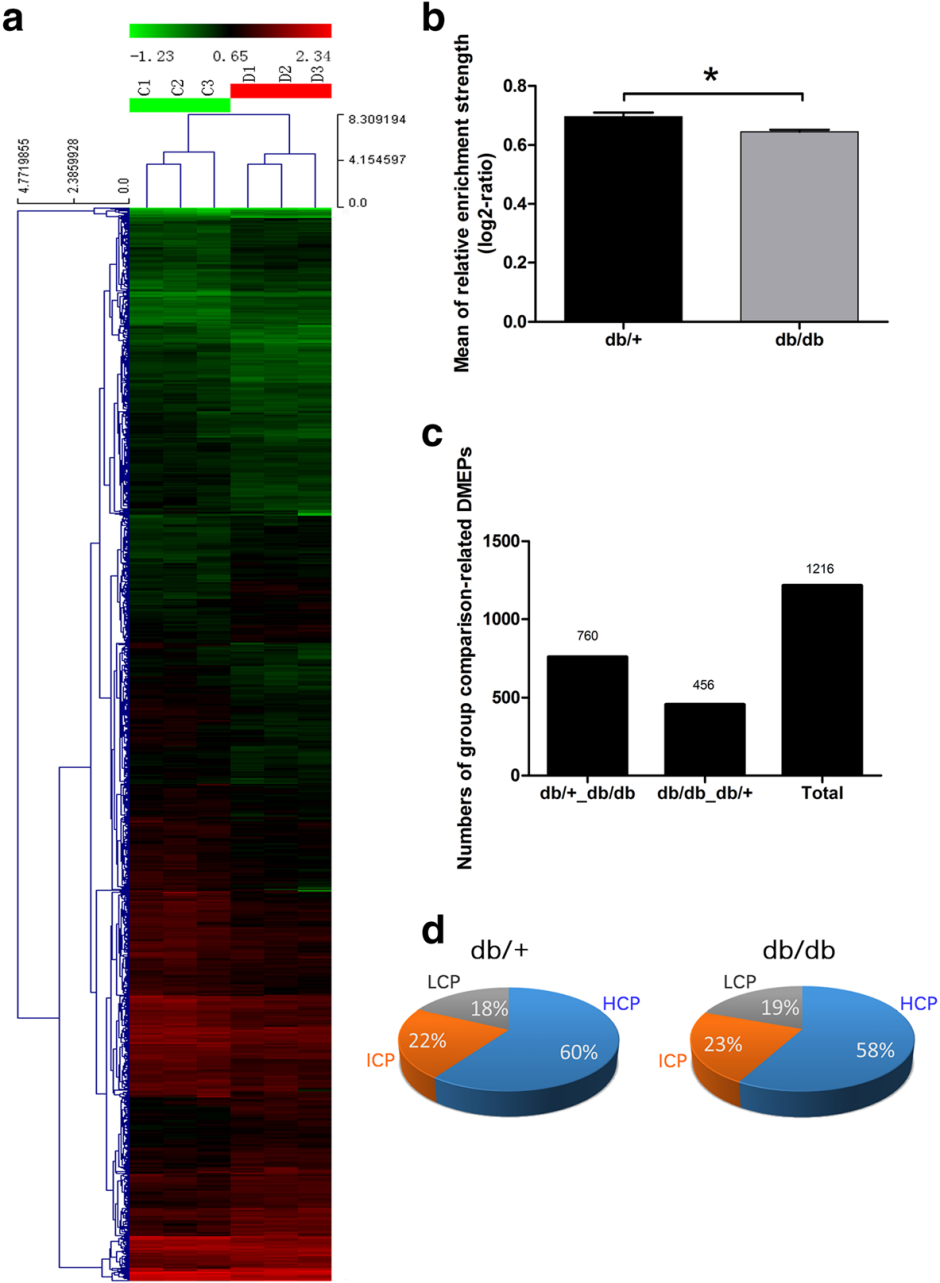
DNA methylation of promoter regions in crypts. **a** Unsupervised hierarchical clustering analysis of DMEPs in crypts. Each *row* indicates a differential methylation enrichment peak (*DMEP*)-related gene. Each *column* indicates a sample analyzed. Methylation levels range from unmethylated (*green*) to fully methylated (*red*), as indicated by the color legend at the top of the graph. *C* represents the control *db/+* group; *D* represents the diabetic *db/db* group. **b** Mean of relative enrichment strength in DMEPs. Mean ± SE; *n* = 3; *P = 0.028 by *t* test. **c** Numbers of group comparison-related DMEPs. *db/+*_*db/db* represents the DMEPs more closely matching the promoter region for the *db/+* group than that in the *db/db* group; and *db/db*_*db/+* represents the DMEPs more closely matching the promoter region for the *db/db* group than that in the *db/+* group. **d** Pie charts of high, intermediate, and low CpG-density promoters (*HCPs*, *ICPs*, and *LCPs*, respectively) indicate slight differences in the percentages of HCPs and LCPs between the two groups

To investigate DNA methylation of promoter regions in abnormal expanded crypts, we used a promoter microarray approach to detect differential methylation of gene promoters in *db/db* and *db/+* mice. Quality assessments of raw data are provided in Additional file 2 (Figure S1). In our study, among the global DNA promoter regions, only approximately 14.1% of promoter regions were methylated (3146 of 22,327; Additional file 1: Table S3). Subsequently, we evaluated the DMEPs (see Methods for statistical method) between the two groups. The total number of DMEPs in the *db/db* mice was less than that in the *db/+* (*P* = 0.028 by *t* test; Fig. 1a and b; Additional file 1: Table S4). To further investigate the DMEPs, we compared the methylated promoters between the two groups. Based on this comparison, we identified a total of 1216 group comparison-related DMEPs, of which 760 were *db/+*_*db/db* (representing DMEPs more closely matching the promoter region for the *db/+* group than that in the *db/db* group) and 456 were *db/db*_*db/+* (representing DMEPs more closely matching the promoter region for the *db/db* group than that in the *db/+* group) (Fig. 1c; Additional file 1: Table S5).

We subdivided promoters into three classes based on the CpG ratio, GC content, and length of the CpG-rich region: high CpG-density promoters (HCPs; promoters that contained a 500 bp interval within −0.7 kb to +0.2 kb with a [G + C]-fraction ≥0.55 and a CpG observed to expected ratio (O/E) ≥0.6), low CpG-density promoters (LCPs; promoters that contained less than 500 bp interval with CpG O/E ≥0.4), and intermediate CpG-density promoters (ICPs; those between HCPs and LCPs). Of the 3146 methylated promoter regions, many regions were HCPs. Only slight differences in the percentages of HCPs or LCPs were identified between the two groups (Fig. 1d).

Taken together, these findings demonstrated decreased DNA methylation of promoter regions in crypts of *db/db* mice; however, the percentages of methylated HCPs, LCPs, or ICPs were not markedly different between the two groups.

### Expression of DNA methyltransferases in the intestinal epithelium was correlated with methylation pattern and diabetic state

**Fig. 2:**
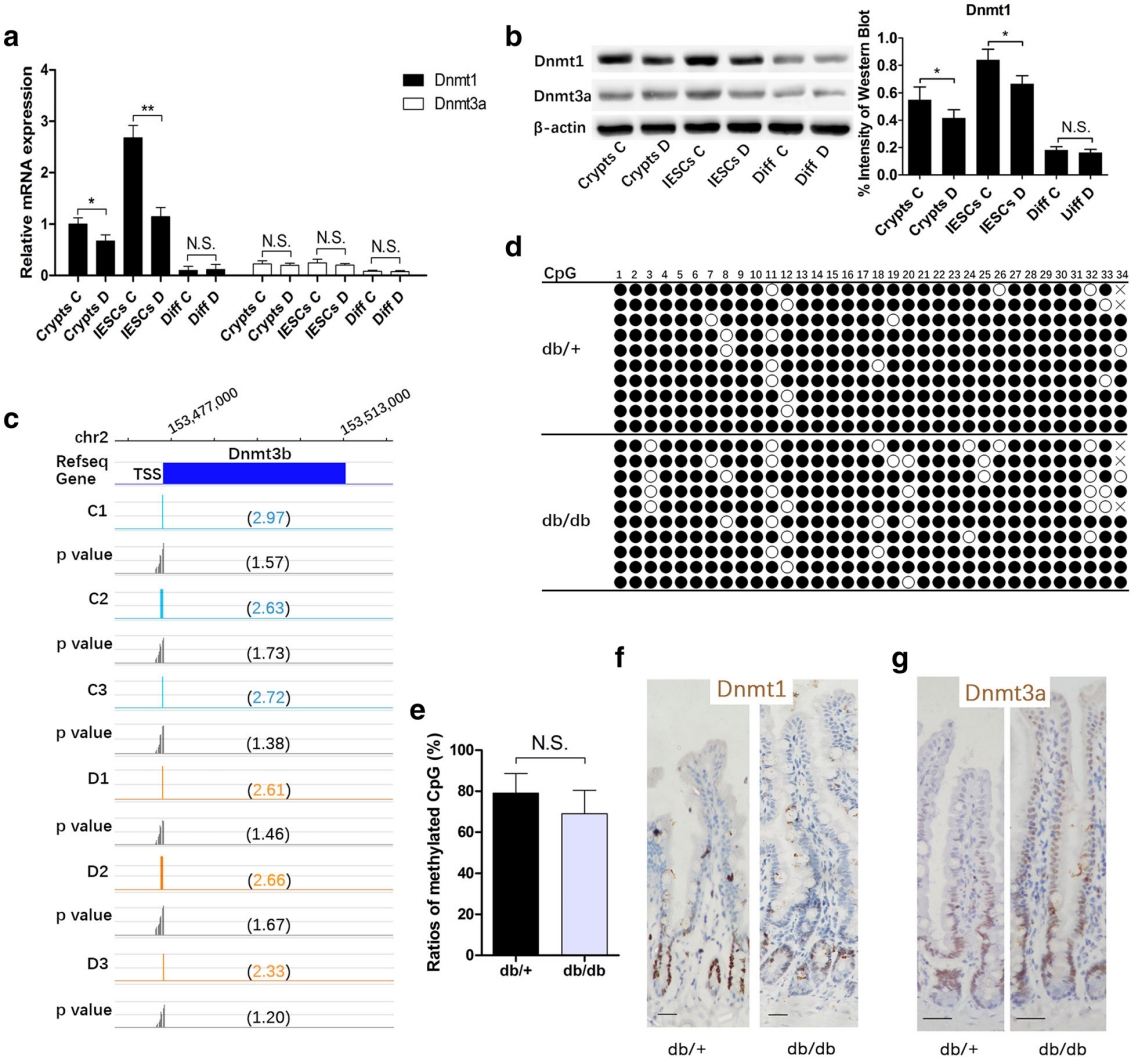
Expression and promoter methylation of DNA methyltransferases in intestinal epithelium. **a, b** Decreased *Dnmt1* expression is detected in *db/db* crypts and intestinal epithelium stem cells (*IESCs*) by qRT-PCR using the FACS cell population (**a**) and Western blot using the MACS cell population (**b** *left*) and quantification of immunoblot bands from Western blot (**b** *right*). mRNA and protein levels are expressed relative to β-actin. Mean ± SE; *n* ≥ 6; *P < 0.05, **P < 0.01 by *t* test. *C* represents the control *db/+* group; *D* represents the diabetic *db/db* group. **c** Promoter methylation microarray of *Dnmt3b* indicates promoter hypermethylation not only in *db/db* mice (*orange bars*) but also in *db/+* mice (*light blue bars*). Values in brackets indicate the peak score that reflects the probability of positive enrichment and average *P* value scores. **d, e** Validation of *Dnmt3b* microarray results by bisulfite sequence. The table indicates the results of bisulfite sequencing analyses of 10 bacterial colonies per group (**d**). The CpG sites validated are the sites that exhibit a peak signal in the microarray. *Black circles* represent methylated CpG sites; *white* hollow circles represent unmethylated CpG sites; *crosses* represents methylation status not determined . Bar graph showing the ratio of methylated cytosine in CpG within the regions analyzed (**e**). **f, g** Immunohistochemical staining of Dnmt1 (**f**) and Dnmt3a (**g**). *Scale bars* = 50 μm. *Diff* differentiated cells, *N.S.* not significant

After obtaining the global DNA methylation of promoter regions in crypts, we investigated three DNA methyltransferases in adult mouse intestines. In our study, *Dnmt1* expression was higher than *Dnmt3a* in the intestinal epithelium (Fig. 2a and b), whereas *Dnmt3b* was minimal and nearly undetected. Based on the microarray results, we speculated that the extremely low level of Dnmt3b might be attributable to the hypermethylation of its promoter (Fig. 2c). Furthermore, validation of the microarray results by bisulfite sequencing also suggested hypermethylation of the *Dnmt3b* promoter, although no difference was identified between the two groups (Fig. 2d and e).

In crypts, significantly lower *Dnmt1* expression was observed in the *db/db* mice compared with the *db/+* mice, whereas no difference in *Dnmt3a* was observed between the two groups. Moreover, a markedly reduced expression of *Dnmt1* was identified in terminally differentiated cells (Fig. 2a and b). Previous studies have reported that Dnmt1 was restricted to the crypts of intestinal epithelium [12, 17]. Similarly, we found that Dnmt1 was limited to the crypt regions in both groups (Fig. 2f), while Dnmt3a exhibited increased expression in crypts but was also expressed in other positions in the epithelium (Fig. 2g).

IESCs serve as the source of all types of enterocytes and perform more prominent proliferation and differentiation activity. Thus, we were interested in the changes in DNA methyltransferases in IESCs. We isolated cells expressing high levels of Lgr5, the genuine stem cells of the small intestine [2], using FACS and MACS (Additional file 3: Figure S2a). Lgr5^hi^CD45^neg^ cells were identified as IESCs. Confirmation of cell purity was performed by qRT-PCR for the stem cell-specific markers Lgr5, CD24, Olfm4, and Ascl2, the enterocyte marker Lct, the endothelial cell marker CD31, and the lymphocyte marker CD45 (Additional file 3: Figure S2b).

Interestingly, *Dnmt1* exhibited distinctly higher expression in Lgr5^hi^CD45^neg^ IESCs than in the crypts as a whole. Moreover, significantly decreased expression of *Dnmt1* was identified in *db/db* mice compared with the *db/+* mice; however, there was still no difference in the level of *Dnmt3a* between the two groups (Fig. 2a and b). These findings suggested that decreased Dnmt1 in *db/db* mice was correlated with the degree to which DNA methylation was reduced. Furthermore, alterations in the DNA methylation pattern and *Dnmt1* expression in crypts may be mostly inherited from IESCs. In fact, reduction of DNA methylation by acute deletion of *Dnmt1* in the intestinal epithelium has been reported to cause crypt expansion in vivo [12].

### Hypomethylation of Wnt signaling was correlated to its overactivation in diabetic *db/db* crypts and IESCs

**Fig. 3:**
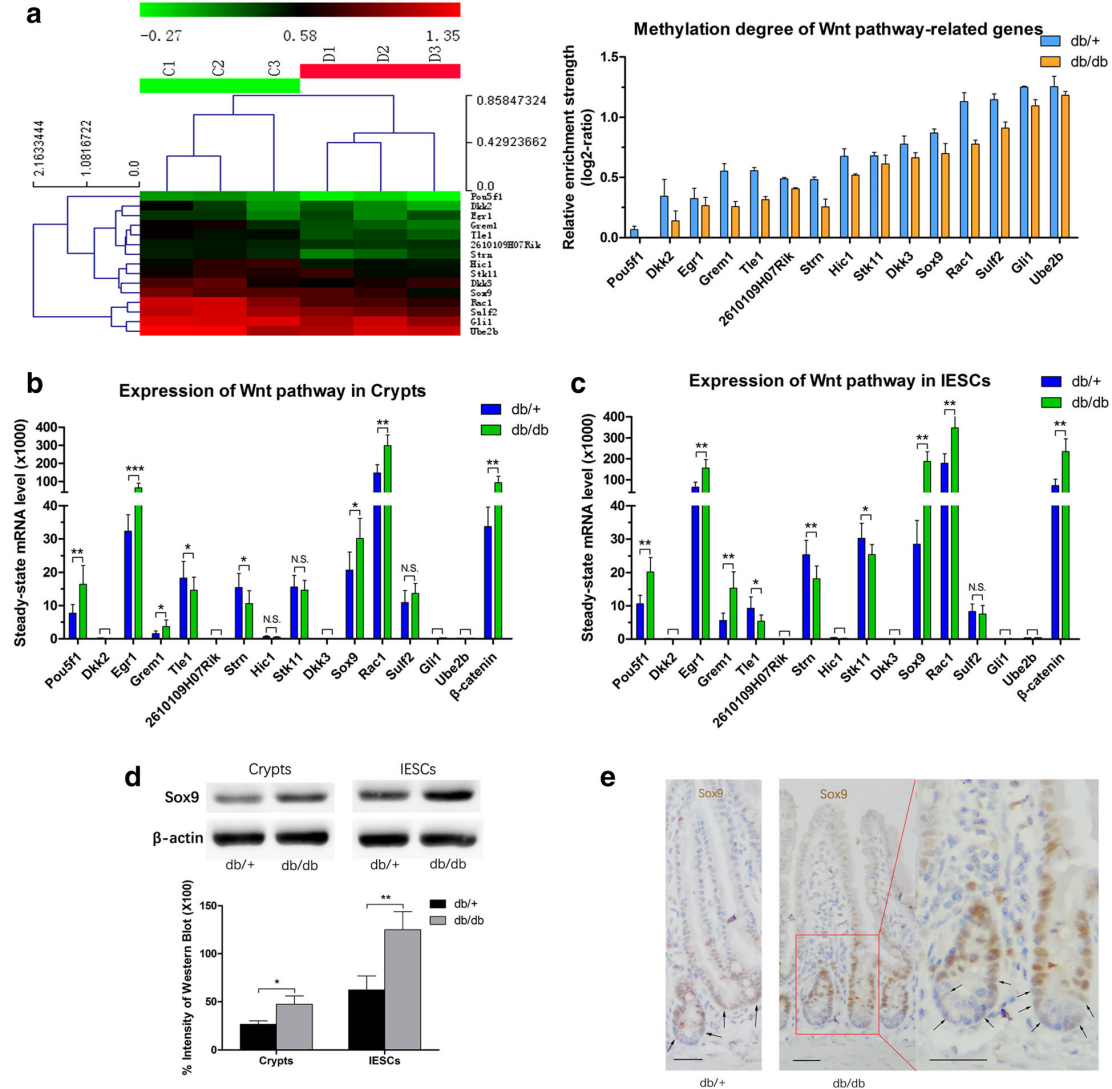
Promoter methylation levels and gene expression of Wnt signaling-related genes. **a** (*left*) Hierarchical clustering analysis on the basis of the DMEP-related genes related with Wnt signaling. Each *row* indicates a DMEP-related gene, and the corresponding gene name is indicated on the *right*. Each *column* indicates a sample analyzed. Methylation levels range from unmethylated (*green*) to fully methylated (*red*), as indicated by the color legend at the top of the graph. **a** (*right*) The degree of methylation of Wnt signaling pathway-related genes among DMEP-related genes. The bar height reflects the degree of methylation. **b, c** The mRNA levels of Wnt signaling-related genes in crypts and intestinal epithelium stem cells (*IESCs*) using the FACS cell population. **d** (*upper*) Western blot analysis of Sox9 using the MACS cell population, and **d** (*lower*) quantification of immunoblot bands from three repetitions of western blot experiments. **e** Immunohistochemical staining of Sox9 in the small intestinal epithelium. The *arrows* indicate cells expressing low levels of Sox9, which were considered IESCs. *Scale bars* = 50 μm. mRNA and protein levels are expressed relative to β-actin. Mean ± SE; *n* ≥ 6 (mRNA); *n* ≥ 3 (protein); *P < 0.05, **P < 0.01, ***P < 0.001 by *t* test. *N.S.* not significant

In our previous study, we demonstrated that enhanced proliferation in the intestinal epithelium of patients with type 2 DM correlated with Wnt/β-catenin accumulation [4]. Wnt signaling was involved in the abnormal proliferation and differentiation of IESCs [2]. Therefore, we focused on the correlation between methylation status and changes in the expression of genes associated with the Wnt signaling cascade in the *db/db* mice. Based on GO analysis and the KEGG (Kyoto Encyclopedia of Genes and Genomes) database, fifteen DMEP-related genes related to Wnt signaling, particularly the canonical Wnt pathway, were identified in the microarray. Indeed, the methylation levels of these genes were decreased in the *db/db* mice (Fig. 3a). Combined with mRNA expression, we identified significant differences between the two groups in the expression of hypomethylated genes, such as *Pou5f1* (POU domain class 5 TF 1), *Egr1* (early growth response 1), *Grem1* (gremlin 1), and *Sox9*, whereas the hypermethylated genes (*Hic1* (hypermethylated in cancer 1), *Stk11* (serine/threonine kinase 11) and *Sulf2* (sulfatase 2)) did not differ between the groups, with the exclusion of *Rac1* (RAS-related C3 botulinum substrate 1; Fig. 3b). Previous studies reported that Rac1 was required for the expansion of the Lgr5-positive IESC signature and for progenitor hyperproliferation [18] and was induced under conditions of intestinal proliferation in response to aberrant Wnt signaling [19]. In our study, Rac1 in both groups was highly expressed despite hypermethylation of its promoter whereas, compared with the control, *Rac1* expression was increased in the *db/db* mice (Fig. 3b). In addition, expression of *Tle1* (transducin-like enhancer of split 1) and *Strn* (striatin) was significantly decreased in the *db/db* mice. Tle1 has been reported to bind to *TCF* to repress the activity of TCF/LEFs [20], and Strn has been reported to colocalize with APC, a tumor suppressor protein that negatively regulated the Wnt signaling pathway [21]. Moreover, *Dkk2* (dickkopf WNT signaling pathway inhibitor 2), *2610109H07Rik* (Draxin, dorsal inhibitory axon guidance protein), *Dkk3* (dickkopf WNT signaling pathway inhibitor 3), *Gli1* (GLI-Kruppel family member 1), and *Ube2b* (ubiquitin-conjugating enzyme E2B) exhibited such extremely low expression in crypts that they could not be used for statistical analysis. In addition to the DMEP-related genes, β-catenin was also evaluated to determine Wnt/β-catenin signaling (Fig. 3b and c).

To further investigate Wnt signaling, we focused on the base of crypts, the IESCs. They were bona fide stem cells that showed fast cycling and were responsive to Wnt signaling [2]. We performed qRT-PCR in IESCs for the DMEP-related genes related to Wnt signaling. Surprisingly, most of the genes, including *Pou5f1*, *Egr1*, *Grem1*, *Strn*, *Stk11*, *Sox9*, and *Rac1*, exhibited increased expression in IESCs compared with crypts (Fig. 3c). One of the most obvious differentially expressed genes between crypts and IESCs was *Sox9*. Increased Sox9 expression was detected in the *db/db* crypts, more remarkably in the *db/db* IESCs (Fig. 3b–d). In fact, Sox9 expression was mainly restrained to the crypts (Fig. 3e) [22], and cells showing low levels of Sox9 were considered IESCs, which were capable of forming cryptoid structures under in vitro culture conditions [23, 24].

Together, these observations indicated that hypomethylation of gene promoters correlated to ectopic Wnt signaling in *db/db* crypts, especially in IESCs. Several TFs, such as Sox9, were also under the regulation of hypomethylation and might cooperate in the control of this aberrant signaling.

### Hypomethylation in the Sox9 promoter was correlated to its overexpression in IESCs

**Fig. 4:**
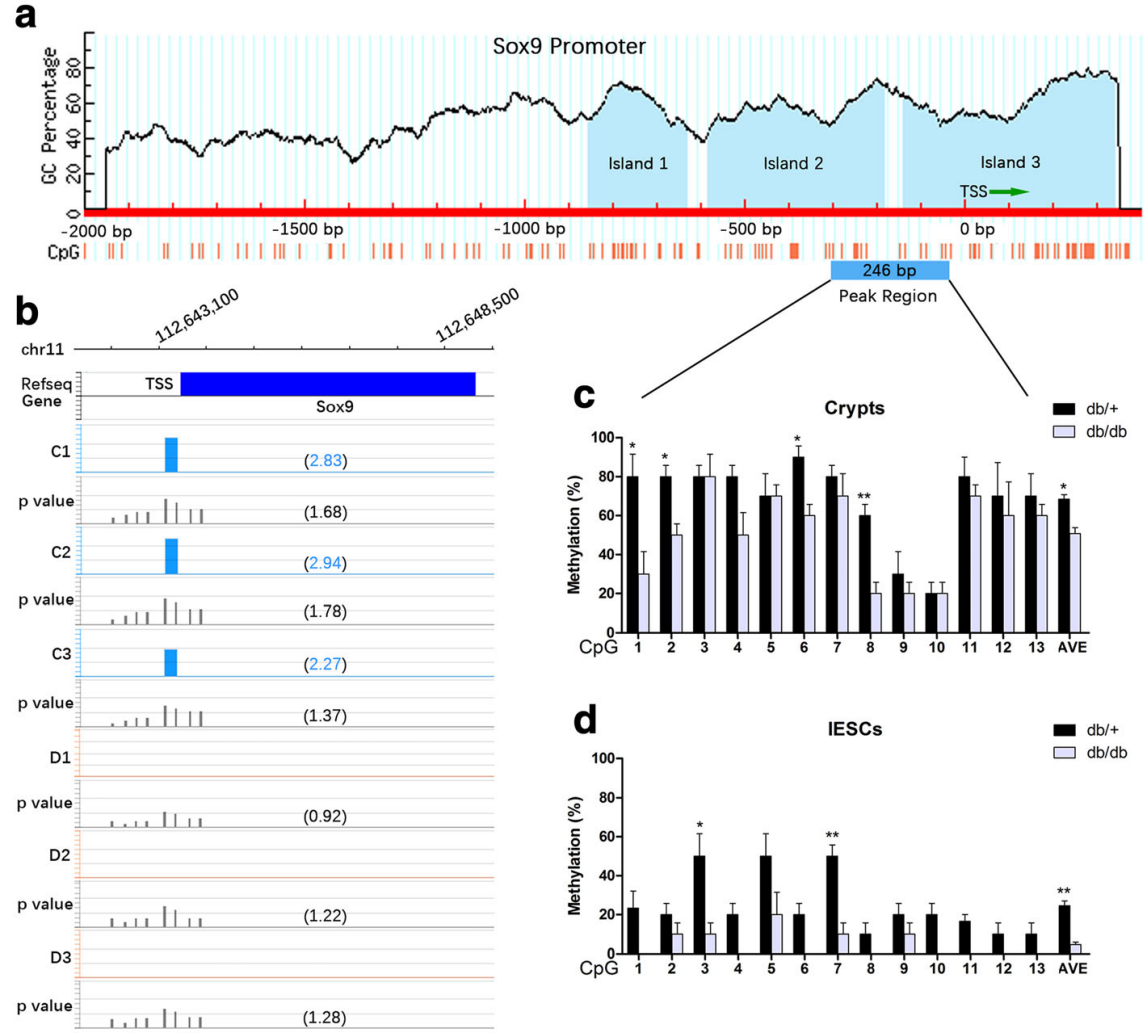
Analyses of CpG islands and methylation of *Sox9*. **a** Structural features of the *Sox9* sequence. The scaled *horizontal line* indicates the genomic DNA sequence, with the transcription start site (*TSS*) at zero; scales represent base pairs. Three CpG islands of *Sox9* close to the TSS are indicated in the *light blue* area on top of the *horizontal line. Vertical lines* beneath the *horizontal line* indicate the distributions and densities of CpG sites. The *light blue rectangular box* indicates the location of the methylation peak signal region detected in the microarray and used in the bisulfite sequence analysis. **b** Promoter methylation microarray of *Sox9* indicating hypermethylated promoters in the *db/+* group (*light blue bars*). Values in brackets indicate the peak score that reflect the probability of positive enrichment and average *P* value scores. **c, d** Validation of *Sox9* microarray results by bisulfite sequencing. Bar graphs indicating the proportion of methylated cytosines in CpGs within the regions analyzed. Ten bacterial colonies are detected per sample. Mean ± SE; *P < 0.05, **P < 0.01 by *t* test. *IESCs* intestinal epithelium stem cells

Among the Wnt signaling pathway-related genes that were also differentially expressed and differentially methylated, we identified Sox9, which is a TF that plays a pivotal role in the regulation of Wnt signaling [7–11] and in the intestinal epithelium [22, 25]. To address whether methylation influenced the differential expression of *Sox9* in IESCs, bisulfite sequencing was examined to investigate DNA methylation of the peak signal region detected in the microarray. The *Sox9* upstream sequence had 3 CpG islands (222 bp, 401 bp, and 482 bp) within 1.3 kb close to the TSS (Fig. 4a). The peak signal region spanned from island 2 to island 3, 246 bp in length, in each *db/+* sample, and it was located from –305 to –59 of the TSS. The range of CpG-rich sequences and their densities were in accordance with classic and default definitions of CpG islands according to the MethPrimer program [26].

In the microarray results, loss of methylation in *Sox9* promoters was detected in the three *db/db* samples (Fig. 4b). Validation by bisulfite sequence analysis revealed that, on average, 50.8% of CpG sites were identified as methylated in the *db/db* mice intestinal crypts compared with 68.5% in the *db/+* (*P* < 0.05; Fig. 4c). However, in IESCs, the methylation status was reduced in both groups, with only 5% in the *db/db* mice and 25% in the *db/+* mice (*P* < 0.01; Fig. 4d). Combined with the gene expression of *Sox9* (Fig. 3b–d), we speculated that hypomethylation in the *Sox9* promoter might contribute to the altered gene expression in diabetic *db/db* crypts. Moreover, the degree of methylation of the *Sox9* promoter was decreased in both groups in IESCs compared with crypts and was markedly decreased in *db/db* IESCs, which in part contributed to the increased Sox9 level.

### Sox9 acted as a transcriptional regulator in Wnt signaling

Sox9 functioned as a TF and regulated gene expression by targeting gene regulatory sequences [8, 10, 11, 27]. We therefore investigated the mechanism by which Sox9 regulated gene expression in *db/db* IESCs. Thus, using FACS-sorted IESCs, we conducted Sox9 ChIP-seq to investigate the interaction of this TF and the genomic DNA sequences in vivo. Quality assessments of chromatin preparation and raw data are provided in Additional file 4 (Figure S3).

**Fig. 5:**
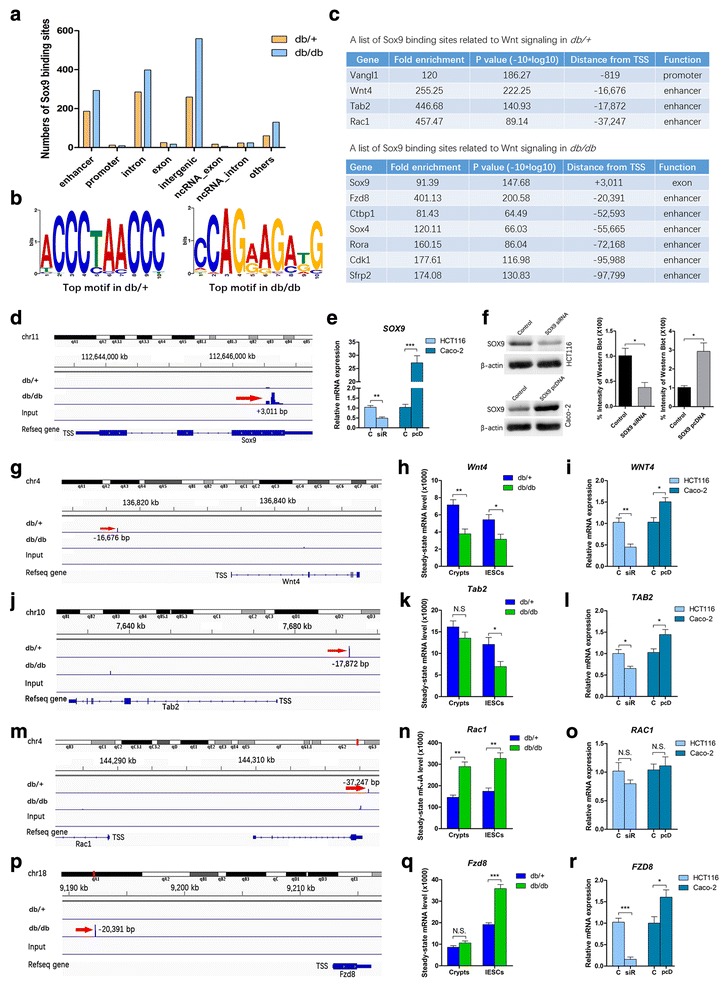
ChIP-seq analysis of Sox9. **a** Distribution of Sox9 binding sites in genetic elements. Many Sox9 binding sites are located in enhancers, introns, and intergenic regions. **b** The most enriched motif within Sox9 signal peaks. **c** A list of Sox9 binding sites related to Wnt signaling. **d, g, j, m, p** Binding profiles and peak calling records of representative Wnt component genes in Sox9 ChIP-seq analysis. *Red arrows* indicate peak signals. **e, f** Knockdown and overexpression of SOX9 in cultured cells. **h, k, n, q** mRNA levels of Sox9 binding site-related genes. **i, l, o, r** mRNA levels of Sox9 binding site-related genes after knockdown and overexpression of SOX9 in cultured cells. mRNA and protein levels are expressed relative to β-actin. Mean ± SE; *n* ≥ 4; *P < 0.05, **P < 0.01, ***P < 0.001 by *t* test. *N.S.* not significant

A total of 1436 Sox9 binding sites were identified in the *db/db* mice, and 867 were identified in the *db/+* mice by Sox9 ChIP-seq (Additional file 1: Table S6). Further analysis of genetic elements of the Sox9 binding sites indicated that most of them were bound to enhancers (−50 kb upstream of and +5 kb downstream from the gene body), introns, and intergenic regions (Fig. 5a). We conducted unbiased sequence motif searches within the Sox9 binding signal peak in the two groups. The top motif in each group indicated a low sequence specificity of Sox9 binding in IESCs (Fig. 5b). Previous studies also demonstrated this sequence characteristic of Sox9 binding in adult hair follicle stem cells [11] and in prostate cancer [10]. Together, these observations suggest a substantial divergence of the Sox9 consensus motif.

To further comprehensively investigate Sox9 binding sites related to the Wnt signaling pathway, we defined a promoter (±2 kb of TSS) and an “extended enhancer” (−100 kb upstream of and +5 kb downstream from the gene body) with reference to previous studies [11, 27]. Subsequently, among the Sox9 binding sites, we identified a list of Wnt signaling pathway-related genes (Fig. 5c). The mRNA level with respect to these genes was detected by qRT-PCR in IESCs and crypts. Moreover, overexpression and knockdown of *SOX9* were performed through an overexpression plasmid in the human colorectal carcinoma cell lines Caco-2 and DLD-1 and through siRNA in HCT116 and HT-29 cells, respectively (Fig. 5e and f).

Among the Wnt signaling pathway-related genes, the mRNA levels of *Vangl1* (vang-like 1), *Rora* (RAR-related orphan receptor alpha), and *Sfrp2* (secreted frizzled-related protein 2) were extremely low in IESCs and crypts (Additional file 5: Figure S4a and b). Thus, we focused on the following eight genes: *Wnt4* (wingless-type MMTV integration site family, member 4), *Tab2* (TGF-beta activated kinase 1/MAP3K7 binding protein 2), *Rac1*, *Sox9*, *Fzd8* (frizzled class receptor 8), *Ctbp1* (C-terminal binding protein-1), *Sox4* (sex determining region Y box 4), and *Cdk1* (cyclin-dependent kinase 1). In a previous study, a *Sox9* far-upstream enhancer located at −70 kb of the TSS was identified; Sox9 dimers directly bound to multiple sites of this sequence and thereby cooperatively activated the enhancer [27]. Surprisingly, in the *db/db* mice, we also discovered a strong peak signal at the third exon of *Sox9*, which was located at +3011 of the TSS (Fig. 5d).

In the *db/+* mice, three enriched peak signals of Sox9 binding sites were located at the enhancers of *Wnt4*, *Tab2*, and *Rac1* (Fig. 5c). Wnt4 was abundantly expressed in β cells in an obese mouse model and acted as an inhibitor of canonical Wnt signaling, as well as an activator of noncanonical Wnt signaling [28]. In our study, the expression of *Wnt4* was decreased in the *db/db* mice, and it was correlated with a lack of the Sox9 binding signal. However, in the *db/+* mice, a peak signal was located at −16,676 bp upstream of TSS (Fig. 5g and h). From the *SOX9* regulation experiments in cultured cells emerged a corresponding positive correlation of mRNA level between SOX9 and WNT4 (Fig. 5i). In addition to WNT4, TAB2 is a putative TAK1 interacting protein that plays an inhibitory role in canonical Wnt/β-catenin signaling [29]. Decreased *Tab2* expression was observed in *db/db* IESCs, and Sox9 targeted the enhancer of *Tab2* in the *db/+* mice at −17,872 bp of the TSS (Fig. 5j and k). The cell experiments also confirmed a positive transcriptional regulation of SOX9 on *TAB2* (Fig. 5l). Another Sox9 binding site in the *db/+* was related to *Rac1*, locating at −37,247 bp upstream of the TSS (Fig. 5m). Although peak signal was also observed in the *db/db* mice, it was eliminated as a ghost signal by the algorithm. Indeed, abundant expression of *Rac1* was identified in crypts and IESCs, and significantly increased *Rac1* expression was observed in the *db/db* mice (Fig. 5n). However, the cell experiments showed no relationship between *Rac1* expression and the Sox9 TF action (Fig. 5o).

In short, these findings indicated that, in normal controls, Sox9 targeted the enhancers of *Wnt4* and *Tab2*, positively regulating gene expression. However, in the *db/db* mice, the expression of *Wnt4* and *Tab2*, both repressors of Wnt signaling, was decreased, and both lacked the Sox9 binding signal. Thus, the lack of Sox9 transcriptional regulation in the repressors of the Wnt signaling pathway may result in abnormalities in this pathway for reducing adequate repressors.

In the *db/db* mice, the strongest enriched peak signal of Sox9 binding sites related to Wnt signaling was located at the enhancer of *Fzd8*, a receptor component in the Wnt pathway, located at −20,391 bp upstream of its TSS (Fig. 5p). There was no significant difference in the expression of *Fzd8* in crypts between the two groups; however, *Fzd8* expression in IESCs was significantly increased in *db/db* mice (Fig. 5q). Moreover, the mRNA level of *FZD8* was dramatically reduced when *SOX9* expression was decreased by siRNA in HCT116 and HT-29 cells. Similarly, the expression of *FZD8* was increased in Caco-2 and DLD-1 cells, accompanied by overexpression of *SOX9* (Fig. 5r). These observations suggested that expression of *Fzd8* was raised by Sox9 transcriptional activation in the *db/db* mice.

In addition, a faint Sox9 binding peak signal in the *db/db* mice was associated with another family member of Sox TFs, Sox4 (Additional file 5: Figure S4c). Sox4 protein stabilized β-catenin and thereby boosted canonical Wnt signaling [30]. We identified increased expression of *Sox4* in *db/db* crypts and IESCs (Additional file 5: Figure S4a and b). Furthermore, the cell experiments also suggested a positive correlation between the mRNA levels of SOX4 and SOX9 (Additional file 5: Figure S4d). This suggested that Sox9 bound to the enhancer of *Sox4* may act as a transcriptional activator. Another weak Sox9 binding peak signal in the *db/db* mice was related to *CtBP1*, a Wnt signaling repressor (Additional file 5: Figure S4e). However, in the cell experiments, there was no regulatory effect (Additional file 5: Figure S4f). Finally, a distant Sox9 binding peak signal was identified at −95,988 bp upstream of the *Cdk1* TSS (Additional file 5: Figure S4g). Since autoregulation of Sox9 by a far-upstream (−70 kb) enhancer was previously demonstrated in somatic tissues [27], distant enhancers may be possible. Intriguingly, we discovered a negative correlation for mRNA levels between SOX9 and CDK1 in cultured cells. Decreased *SOX9* expression markedly increased the *CDK1* level, whereas overexpression of *SOX9* limited the expression of *CDK1* (Additional file 5: Figure S4h). This suggested that SOX9 inhibited the transcription of *CDK1*. These findings were in contrast to the previously described results showing the function of Sox9 as a transcriptional activator.

**Fig. 6:**
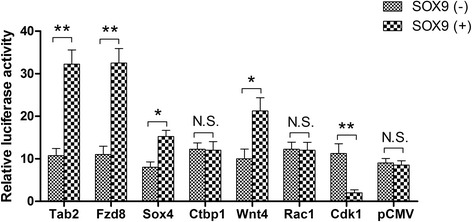
Relative luciferase activities driven by sequences encompassing Sox9 ChIP-seq binding sites. Results are presented as the enhancement of luciferase activity in the presence of a SOX9 expression vector (SOX9 (+)) compared with an empty vector (SOX9 (−)). All results are normalized to the luciferase activity of Renilla luciferase control reporter vector. Experiments for each construct were performed in four replicates in duplicate wells. Mean ± SE; *P < 0.05, **P < 0.01 by *t* test . *N.S.* not significant

To further validate that Sox9 regulates the transcription of these genes, we carried out luciferase reporter assays in HEK 293FT cells with or without SOX9 expression vector. Relative to the control minimal CMV promoter, the Sox9 binding sites of either *Tab2*, *Fzd8*, *Sox4*, *Ctbp1*, *Wnt4I,* or *Rac1* displayed increased enhancer activity with the SOX9 expression vector (Fig. 6). However, the Sox9 binding site of *Cdk1* displayed decreased enhancer activity with the SOX9 expression vector. The effects were SOX9-dependent since significantly decreased enhancer activities were detected in these Sox9 binding sites with the SOX9 empty vector (Fig. 6). These data establish *Tab2*, *Fzd8*, *Sox4*, *Wnt4*, and *Cdk1* as bona fide targets of SOX9.

Taken together, these observations suggested that Sox9 targeted the enhancers of specific Wnt signaling-related genes. Sox9 was not only predominantly acting as a transcriptional activator at proximal enhancers (*Tab2*, *Fzd8*, *Sox4*, and *Wnt4*) but was also acting as a potential transcriptional inhibitor at a distant enhancer (*Cdk1*). The lack of Sox9 transcriptional activation of specific repressors of the Wnt signaling pathway contributed to the formation of abnormal activity of this pathway in *db/db* mice.

## Discussion

We identified hypomethylated promoters and overactivated Wnt signaling in intestinal crypts in diabetic *db/db* mice. Moreover, the expression of *Dnmt1* was decreased in *db/db* crypts, especially in IESCs. Consequently, hypomethylation that contributes to aberrant proliferation and differentiation in the diabetic intestinal epithelium may exist in IESCs. The hypomethylation pattern of promoters in crypts may derive from the methylation status of IESCs. Although dynamic DNA methylation during IESC differentiation has been recognized, the degree of this dynamic process has not yet reached consensus [12, 14, 31, 32]. Nevertheless, there is no doubt that the epigenetic characteristics of IESCs can be at least partially passed on to their daughter cells. Low methylation of Wnt-related genes is observed in IESCs and progenitor cells in *Dnmt1*-deficient mice [12]. Moreover, we detected overactivated Wnt signaling in diabetic *db/db* mice not only in crypts but also in IESCs, particularly significantly in the latter. Further evidence is required to demonstrate the methylation connection between IESCs and their daughter cells in the intestinal epithelium. We consider that reduced DNA methylation in promoters driving gene expression participates in the aberrant proliferation and differentiation in intestinal epithelium due to overactivated Wnt signaling.

Of the differentially methylated genes related to Wnt signaling, we focused on Sox9. Sox9 is known as a Wnt-responsive IESC gene marker [22–24]. Furthermore, Sox9 functions as a TF, regulating gene expression. Nevertheless, the behavior of Sox9 in Wnt signaling remains controversial. On the one hand, in HEK 293 cells, Sox9 inhibits the activation of β-catenin-dependent promoters and stimulates the degradation of β-catenin. Similarly, in Xenopus embryos, Sox9 inhibits the β-catenin-mediated induction of the secondary axis [7]. In chondrocyte differentiation, the Sox9 N-terminus promotes β-catenin degradation, whereas the C-terminus inhibits β-catenin transcriptional activity without affecting its stability. Nuclear localization of Sox9 enhances β-catenin phosphorylation and its subsequent degradation [33]. In cancer cells, Sox9 and Krüppel-like factor 4 (KLF4) interact with β-catenin and reduce its binding to TCF4 [34] and showed anti-oncogenic activity by inhibiting the activity of the Wnt/β-catenin signaling pathway [35]. These reports suggest that Sox9 serves as a negative transcriptional regulator of Wnt signaling. On the other hand, recent ChIP studies have demonstrated a direct correlation between Sox9 and Wnt signaling. In hepatocellular carcinoma, Sox9 activates the canonical Wnt pathway through direct binding with *Fzd7* [9]. Analyses of prostate cancer on xenografts and clinical samples both reveal that SOX9 positively regulates multiple WNT pathway genes, including frizzled (*FZD*), lipoprotein receptor-related protein (*LRP*) family members, and the downstream β-catenin effector *TCF4* [10]. Furthermore, in colon carcinoma cells, an active β-catenin-TCF4 complex is required for physiological targeting of SOX9 [36]. One study suggests a bimodal role for Sox9 in the intestinal epithelium, in which a low level of Sox9 supports proliferative capacity through Wnt/β-catenin signaling, whereas a high level of Sox9 suppresses this process [23]. In a *Sox9* knockout mouse model, Wnt target genes appear to be unaltered in telogen hair follicles [11].

Most previous reports concerned with Sox9 functions on Wnt signaling are focused on cancer cells or embryonic cells. Notably, these are rapidly cycling cells, some of which are outside the normal regulation of the organism. Moreover, embryonic cells are also involved in developmental mechanisms. Thus, it is difficult to use them to represent the condition of adult stem cells. IESCs are bona fide stem cells that are fast cycling and responsive to Wnt signaling [2], which seems to be similar to cancer cells. The intestinal epithelial cells are renewed every 4–5 days. However, these rapid physiological processes of proliferation and differentiation related to Wnt signaling are under intricate control by the organism, requiring multiple intrinsic inhibitors of this signaling. In our research, we specifically consider the influence of methylation on *Sox9* expression in IESCs. In crypts, the *db/db* mice show a lower degree of methylation of *Sox9*, which is accompanied by increased gene expression. In IESCs, promoter methylation of *Sox9* in both groups is significantly reduced, especially in diabetic *db/db* IESCs. The loss of promoter methylation of *Sox9* might induce its prominently increased expression in diabetic *db/db* IESCs. Thus, we consider that the differential expression of *Sox9* in diabetic *db/db* IESCs results in part from the loss of methylation in its promoter. Sox9 is predominately expressed in crypts, and cells that express “low” levels of Sox9 in crypts are considered bona fide IESCs [23–25]. IESCs represent a small percentage of the total crypt cells; however, these cells present prominent proliferation and differentiation in response to Wnt signaling.

Combining ChIP-seq on Sox9 in IESCs with overexpression and knockdown of *SOX9* in cultured cells, we demonstrate that, among Sox9 binding sites related to Wnt signaling, Sox9 targets the enhancers of Wnt signaling pathway-related genes in IESCs, acting not only as a transcriptional activator at proximal enhancers, such as the enhancers of *Wnt4*, *Tab2*, *Sox4*, and *Fzd8*, but also as a potential transcriptional inhibitor at a distant enhancer, such as the enhancer of *Cdk1*. More significantly, lack of Sox9 transcriptional activation of specific repressors of Wnt signaling pathway in the *db/db* mice contributes to the development of abnormal activity of this pathway. Therefore, the overactivated Wnt signaling pathway in *db/db* IESCs might not result directly from Sox9 transcriptional activation of Wnt signaling; rather, it might be a result of losing the intrinsic inhibitory action of repressors.

Notably, in addition to Sox9, other regulators of Wnt signaling may exist in IESCs. Our current study provides a unique research method called the net-to-net method on an abnormal state. An organism is a complex and integrated organic system that can be considered as similar to a multinetwork. Alteration of each point may impact one or more networks. We performed our study using a high-throughput methylation microarray of whole genomic promoters (starting from one network) and subsequently determined the key regulator (here, Sox9) that more or less affects the entire network. Next, we investigated the network changed by the key regulator by using another round of high-throughput sequencing (to another network). We provide a distinctive research perspective on the connections of DNA methylation, TF modulation, and Wnt signaling in IESCs in an animal model of type 2 DM, a common disease with a high risk of transformation to cancer [37].

## Conclusions

Our study sheds light on the connections among DNA methylation, TF modulation, and Wnt signaling in IESCs in the diabetic state. Hypomethylation in the Sox9 promoter is correlated with increased *Sox9* gene expression in *db/db* IESCs. Although *db/db* IESCs showed increased expression of *Sox9*, the loss of Sox9 transcriptional activation in specific repressors of the Wnt signaling pathway might result in abnormal activity of this pathway.

## Additional files

Additional file 1: Table S1. qRT-PCR primer sequences. **Table S2.** Bisulfite-sequencing primer sequences. **Table S3.** Summary of methylation enrichment peaks (MEPs) promoter regions. **Table S4.** Unsupervised hierarchical clustering analysis of DMEPs. **Table S5.** Summary of group comparison-related DMEPs. **Table S6.** Sox9 binding peak signal by ChIP-seq. (XLSX 934 kb)
Additional file 2: Figure S1. Quality assessment of raw data for promoter methylation analysis. (a) MA plot showing the distribution of the red/green intensity ratio (‘M’) plotted based on the average intensity (‘A’). (b) Correlation matrix describing the correlations among replicate experiments. C represents the control *db/+* group; D represents the diabetic *db/db* group. (TIF 5070 kb)
Additional file 3: Figure S2. FACS purification strategy to isolate IESCs and validate cell purity by qRT-PCR. (a) IESCs were isolated by FACS using antibodies against Lgr5 and CD45. Heteromorphic cells and fragments are eliminated by Forward Scatter (FSC) and Side Scatter (SSC). Then, CD45(–) and eflour660 are used to select CD45^neg^ cells, and eventually cells with high Lgr5 expression are used. (b) Validation of cell purity by qRT-PCR. Four cell populations were obtained by FACS (IESCs-FACS), MACS (IESCs-MACS) and EDTA physical isolation technology (Crypts and Diff (differentiated cells)). * represents the comparison between IESCs-FACS and Crypts; ^#^ represents the comparison between IESCs-MACS and Crypts. mRNA levels are expressed relative to β-actin. Mean ± SE; *n* ≥ 6; *, ^#^ *P* < 0.05 by *t* test. (TIF 2091 kb)
Additional file 4: Figure S3. Quality assessments of chromatin preparation for ChIP-seq analysis. (a) Counts of filtered raw data. (b) Distributions of base pairs after filtration. Green area indicates high-quality base pairs; yellow area indicates moderate-quality base pairs; red area indicates poor-quality base pairs. (TIF 4382 kb)
Additional file 5: Figure S4. Sox9 acts as a transcriptional regulator in Wnt signaling. (a and b) Expression of Sox9 binding site-related genes in crypts (a) and IESCs (b). (c, e, and g) Binding profiles and peak calling records of representative Wnt component genes in Sox9 ChIP-seq analysis. Red arrows indicate peak signals. (d, f, and h) mRNA levels of Sox9 binding site-related genes after knockdown and overexpression of *SOX9* in cultured cells. mRNA levels are expressed relative to β-actin. Mean ± SE; *n* ≥ 4; **P* < 0.05, ***P* < 0.01, ****P* < 0.001 by *t* test. *N.S.* not significant. (TIF 4817 kb)

## Abbreviations

ChIP: Chromatin immunoprecipitation
ChIP-seq: Chromatin immunoprecipitation combined with deep sequencing
DM: Diabetes mellitus
DMEP: Differential methylation enrichment peak
Dnmt1: DNA methyltransferase 1
FACS: Fluorescence-activated cell sorting
HCP: High-CpG-density promoter
ICP: Intermediate-CpG-density promoter
IESC: Intestinal epithelium stem cell
LCP: Low-CpG-density promoter
Lgr5: Leucine-rich repeat-containing G protein-coupled receptor 5
MACS: Magnetic bead-activated cell sorting
qRT-PCR: Quantitative real-time polymerase chain reaction
Sox9: Sry (sex determining region Y)-box 9
TF: Transcription factor
TSS: Transcription start site

## References

[1] Min XH, Yu T, Qing Q, Yuan YH, Zhong W, Chen GC (2014). Abnormal differentiation of intestinal epithelium and intestinal barrier dysfunction in diabetic mice associated with depressed Notch/NICD transduction in Notch/Hes1 signal pathway. Cell Biol Int.

[2] Barker N, van Es JH, Kuipers J, Kujala P, van den Born M, Cozijnsen M (2007). Identification of stem cells in small intestine and colon by marker gene Lgr5. Nature.

[3] Zhong XY, Yu T, Zhong W, Li JY, Xia ZS, Yuan YH (2015). Lgr5 positive stem cells sorted from small intestines of diabetic mice differentiate into higher proportion of absorptive cells and Paneth cells in vitro. Dev Growth Differ.

[4] Li JY, Yu T, Xia ZS, Chen GC, Yuan YH, Zhong W (2014). Enhanced proliferation in colorectal epithelium of patients with type 2 diabetes correlates with beta-catenin accumulation. J Diabetes Complications.

[5] Dorfman T, Pollak Y, Sohotnik R, Coran AG, Bejar J, Sukhotnik I (2015). Enhanced intestinal epithelial cell proliferation in diabetic rats correlates with beta-catenin accumulation. J Endocrinol.

[6] Barker N, Ridgway RA, van Es JH, van de Wetering M, Begthel H, van den Born M (2009). Crypt stem cells as the cells-of-origin of intestinal cancer. Nature.

[7] Akiyama H, Lyons JP, Mori-Akiyama Y, Yang X, Zhang R, Zhang Z (2004). Interactions between Sox9 and beta-catenin control chondrocyte differentiation. Genes Dev.

[8] Bastide P, Darido C, Pannequin J, Kist R, Robine S, Marty-Double C (2007). Sox9 regulates cell proliferation and is required for Paneth cell differentiation in the intestinal epithelium. J Cell Biol.

[9] Leung CO, Mak WN, Kai AK, Chan KS, Lee TK, Ng IO (2016). Sox9 confers stemness properties in hepatocellular carcinoma through Frizzled-7 mediated Wnt/beta-catenin signaling. Oncotarget.

[10] Ma F, Ye H, He HH, Gerrin SJ, Chen S, Tanenbaum BA (2016). SOX9 drives WNT pathway activation in prostate cancer. J Clin Invest.

[11] Kadaja M, Keyes BE, Lin M, Pasolli HA, Genander M, Polak L (2014). SOX9: a stem cell transcriptional regulator of secreted niche signaling factors. Genes Dev.

[12] Sheaffer KL, Kim R, Aoki R, Elliott EN, Schug J, Burger L (2014). DNA methylation is required for the control of stem cell differentiation in the small intestine. Genes Dev.

[13] Elliott EN, Sheaffer KL, Schug J, Stappenbeck TS, Kaestner KH (2015). Dnmt1 is essential to maintain progenitors in the perinatal intestinal epithelium. Development.

[14] Yu DH, Gadkari M, Zhou Q, Yu S, Gao N, Guan Y (2015). Postnatal epigenetic regulation of intestinal stem cells requires DNA methylation and is guided by the microbiome. Genome Biol.

[15] Kobayashi K, Forte TM, Taniguchi S, Ishida BY, Oka K, Chan L (2000). The db/db mouse, a model for diabetic dyslipidemia: molecular characterization and effects of Western diet feeding. Metabolism.

[16] Zhang Y, Liu T, Meyer CA, Eeckhoute J, Johnson DS, Bernstein BE (2008). Model-based analysis of ChIP-Seq (MACS). Genome Biol.

[17] Elliott EN, Sheaffer KL, Kaestner KH. The ‘de novo’ DNA methyltransferase Dnmt3b compensates the Dnmt1-deficient intestinal epithelium. Elife. 2016;5. doi: 10.7554/eLife.12975 10.7554/eLife.12975 PMC4786433 26808831

[18] Myant KB, Cammareri P, McGhee EJ, Ridgway RA, Huels DJ, Cordero JB (2013). ROS production and NF-kappaB activation triggered by RAC1 facilitate WNT-driven intestinal stem cell proliferation and colorectal cancer initiation. Cell Stem Cell.

[19] Cheung EC, Lee P, Ceteci F, Nixon C, Blyth K, Sansom OJ (2016). Opposing effects of TIGAR- and RAC1-derived ROS on Wnt-driven proliferation in the mouse intestine. Genes Dev.

[20] Chodaparambil JV, Pate KT, Hepler MR, Tsai BP, Muthurajan UM, Luger K (2014). Molecular functions of the TLE tetramerization domain in Wnt target gene repression. EMBO J.

[21] Breitman M, Zilberberg A, Caspi M, Rosin-Arbesfeld R (2008). The armadillo repeat domain of the APC tumor suppressor protein interacts with Striatin family members. Biochim Biophys Acta.

[22] Furuyama K, Kawaguchi Y, Akiyama H, Horiguchi M, Kodama S, Kuhara T (2011). Continuous cell supply from a Sox9-expressing progenitor zone in adult liver, exocrine pancreas and intestine. Nat Genet.

[23] Formeister EJ, Sionas AL, Lorance DK, Barkley CL, Lee GH, Magness ST (2009). Distinct SOX9 levels differentially mark stem/progenitor populations and enteroendocrine cells of the small intestine epithelium. Am J Physiol Gastrointest Liver Physiol.

[24] Gracz AD, Ramalingam S, Magness ST (2010). Sox9 expression marks a subset of CD24-expressing small intestine epithelial stem cells that form organoids in vitro. Am J Physiol Gastrointest Liver Physiol.

[25] Roche KC, Gracz AD, Liu XF, Newton V, Akiyama H, Magness ST (2015). SOX9 maintains reserve stem cells and preserves radioresistance in mouse small intestine. Gastroenterology.

[26] Li LC, Dahiya R (2002). MethPrimer: designing primers for methylation PCRs. Bioinformatics.

[27] Mead TJ, Wang Q, Bhattaram P, Dy P, Afelik S, Jensen J (2013). A far-upstream (–70 kb) enhancer mediates Sox9 auto-regulation in somatic tissues during development and adult regeneration. Nucleic Acids Res.

[28] Krutzfeldt J, Stoffel M (2010). Regulation of wingless-type MMTV integration site family (WNT) signalling in pancreatic islets from wild-type and obese mice. Diabetologia.

[29] Ishitani T, Ninomiya-Tsuji J, Nagai S, Nishita M, Meneghini M, Barker N (1999). The TAK1-NLK-MAPK-related pathway antagonizes signalling between beta-catenin and transcription factor TCF. Nature.

[30] Lien WH, Polak L, Lin M, Lay K, Zheng D, Fuchs E (2014). In vivo transcriptional governance of hair follicle stem cells by canonical Wnt regulators. Nat Cell Biol.

[31] Huang CZ, Yu T, Chen QK (2015). DNA methylation dynamics during differentiation, proliferation, and tumorigenesis in the intestinal tract. Stem Cells Dev.

[32] Kaaij LT, van de Wetering M, Fang F, Decato B, Molaro A, van de Werken HJ (2013). DNA methylation dynamics during intestinal stem cell differentiation reveals enhancers driving gene expression in the villus. Genome Biol.

[33] Topol L, Chen W, Song H, Day TF, Yang Y (2009). Sox9 inhibits Wnt signaling by promoting beta-catenin phosphorylation in the nucleus. J Biol Chem.

[34] Sellak H, Wu S, Lincoln TM (2012). KLF4 and SOX9 transcription factors antagonize beta-catenin and inhibit TCF-activity in cancer cells. Biochim Biophys Acta.

[35] Prevostel C, Rammah-Bouazza C, Trauchessec H, Canterel-Thouennon L, Busson M, Ychou M (2016). SOX9 is an atypical intestinal tumor suppressor controlling the oncogenic Wnt/β-catenin signaling. Oncotarget.

[36] Blache P, van de Wetering M, Duluc I, Domon C, Berta P, Freund JN (2004). SOX9 is an intestine crypt transcription factor, is regulated by the Wnt pathway, and represses the CDX2 and MUC2 genes. J Cell Biol.

[37] Giovannucci E, Harlan DM, Archer MC, Bergenstal RM, Gapstur SM, Habel LA (2010). Diabetes and cancer: a consensus report. CA Cancer J Clin.

