# Supplementary figures and images for: Sox9 transcriptionally regulates Wnt signaling in intestinal epithelial stem cells in hypomethylated crypts in the diabetic state
Source: Stem Cell Res Ther. 2017 Mar 9;8:60. doi: 10.1186/s13287-017-0507-4 (PMC5345140; doi:10.1186/s13287-017-0507-4)

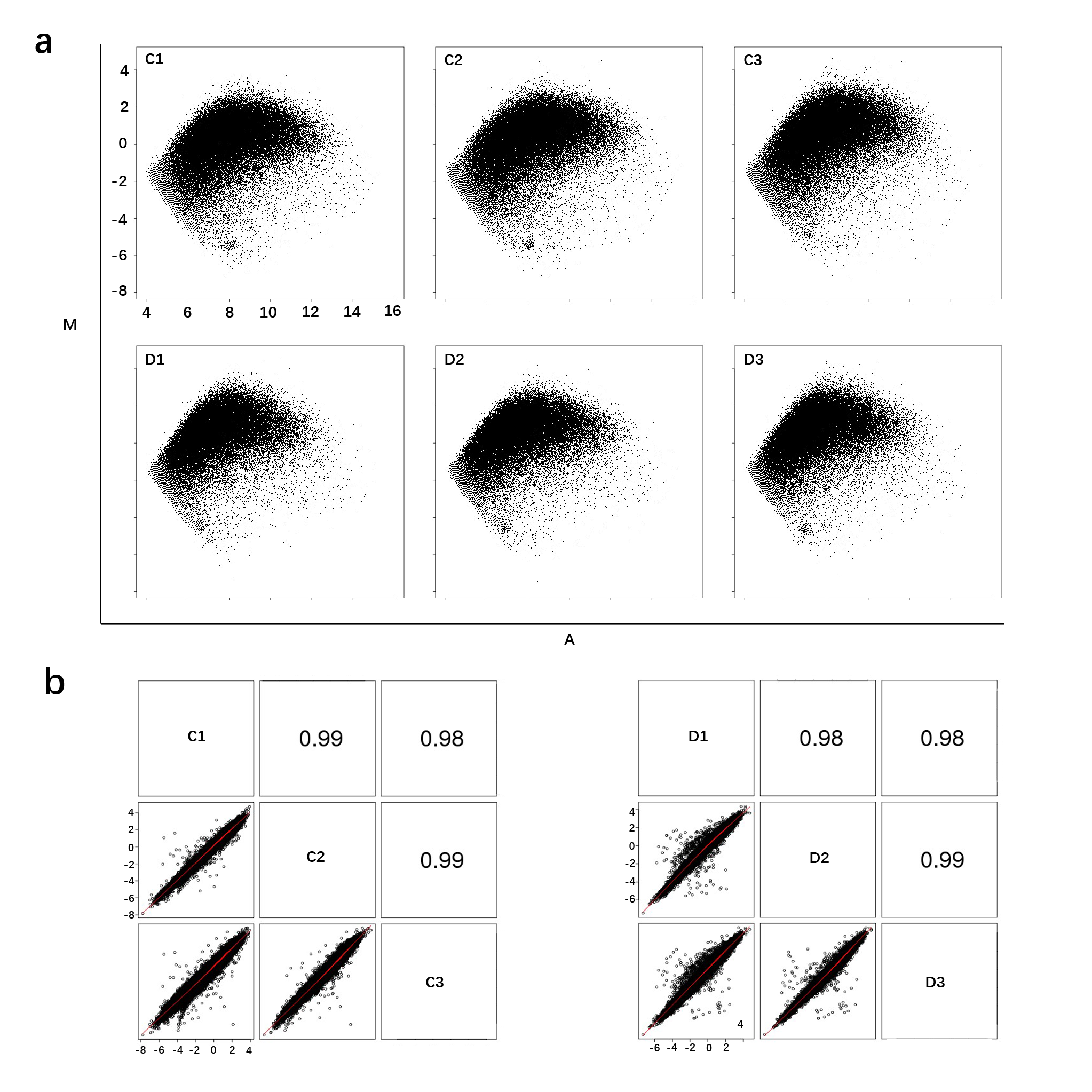

Supplement: Additional file 2: Figure S1. — Quality assessment of raw data for promoter methylation analysis. (a) MA plot showing the distribution of the red/green intensity ratio (‘M’) plotted based on the average intensity (‘A’). (b) Correlation matrix describing the correlations among replicate experiments. C represents the control db/+ group; D represents the diabetic db/db group. (TIF 5070 kb) [file 13287_2017_507_MOESM2_ESM.tif]

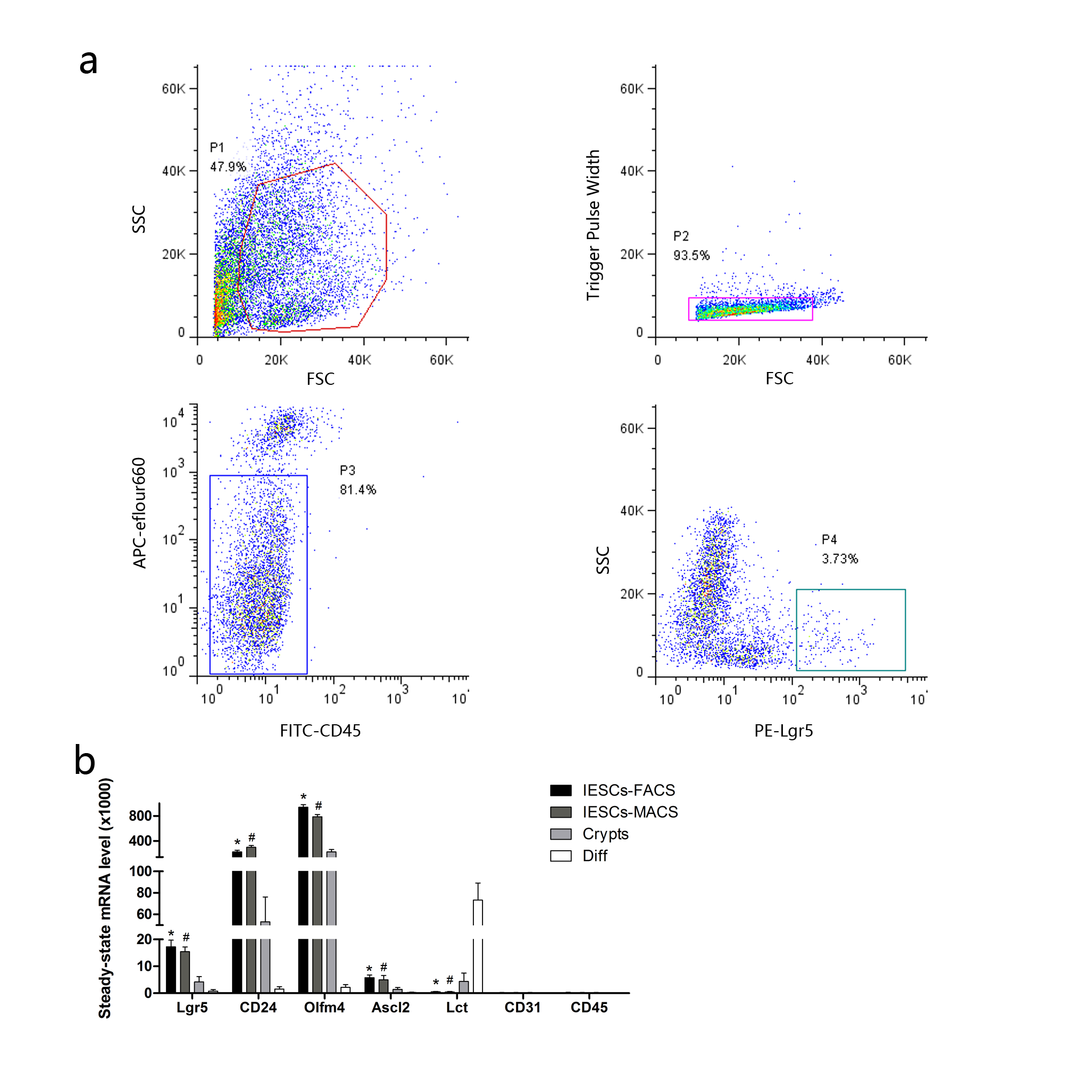

Supplement: Additional file 3: Figure S2. — FACS purification strategy to isolate IESCs and validate cell purity by qRT-PCR. (a) IESCs were isolated by FACS using antibodies against Lgr5 and CD45. Heteromorphic cells and fragments are eliminated by Forward Scatter (FSC) and Side Scatter (SSC). Then, CD45(–) and eflour660 are used to select CD45neg cells, and eventually cells with high Lgr5 expression are used. (b) Validation of cell purity by qRT-PCR. Four cell populations were obtained by FACS (IESCs-FACS), MACS (IESCs-MACS) and EDTA physical isolation technology (Crypts and Diff (differentiated cells)). * represents the comparison between IESCs-FACS and Crypts; # represents the comparison between IESCs-MACS and Crypts. mRNA levels are expressed relative to β-actin. Mean ± SE; n ≥ 6; *, # P < 0.05 by t test. (TIF 2091 kb) [file 13287_2017_507_MOESM3_ESM.tif]

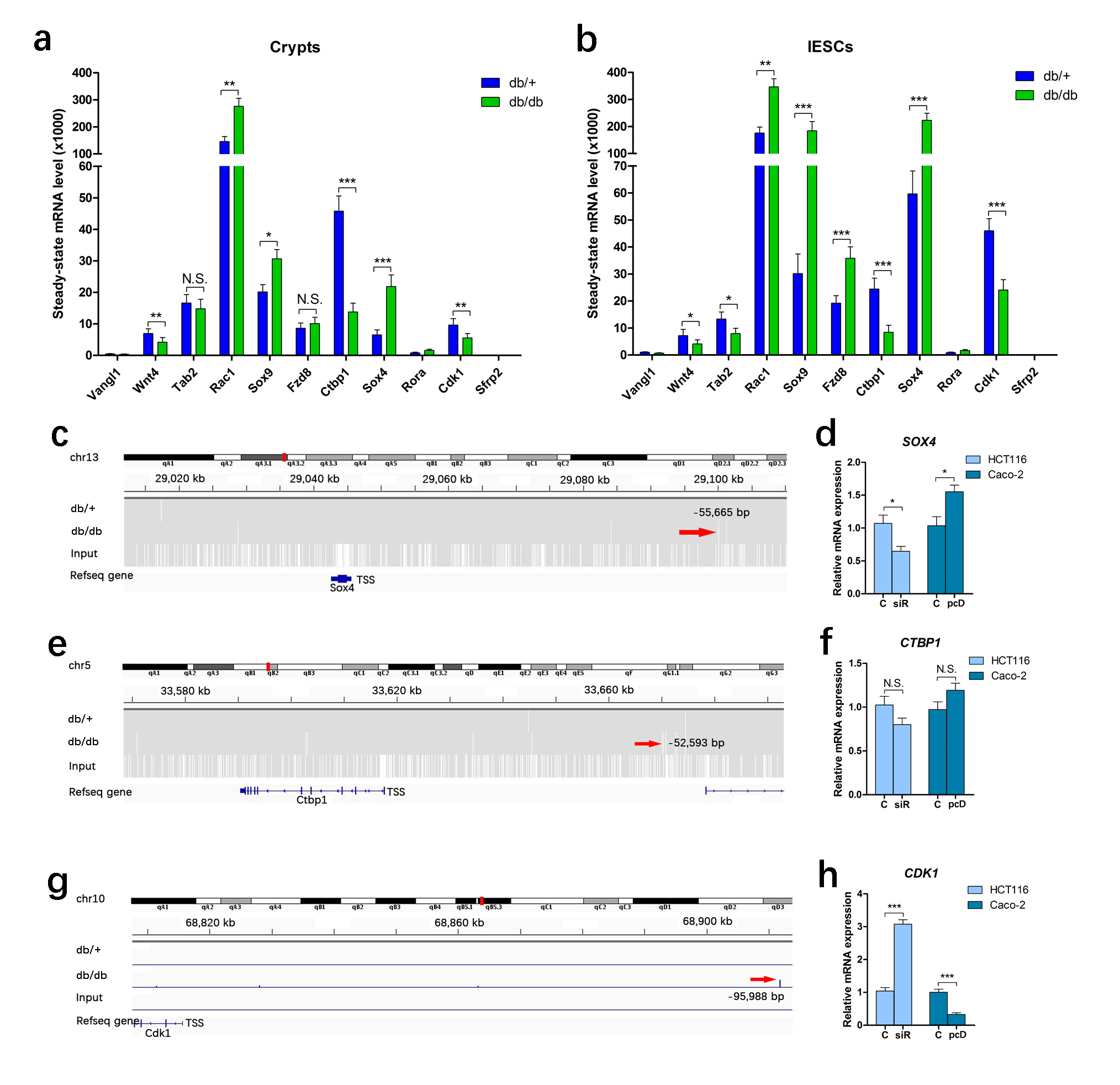

Supplement: Additional file 4: Figure S3. — Quality assessments of chromatin preparation for ChIP-seq analysis. (a) Counts of filtered raw data. (b) Distributions of base pairs after filtration. Green area indicates high-quality base pairs; yellow area indicates moderate-quality base pairs; red area indicates poor-quality base pairs. (TIF 4382 kb) [file 13287_2017_507_MOESM4_ESM.tif]
